# MMP/TIMP expression profiles in distinct lung disease models: implications for possible future therapies

**DOI:** 10.1186/1465-9921-10-72

**Published:** 2009-08-03

**Authors:** Sissie Wong, Maria G Belvisi, Mark A Birrell

**Affiliations:** 1 Respiratory Pharmacology Group, Airways Disease Section, Imperial College London, Faculty of Medicine, National Heart and Lung Institute, 1^st ^Floor - Room 102, Sir Alexander Fleming Building, South Kensington Campus, Exhibition Road, London, SW7 2AZ, U.K

## Abstract

**Background:**

There is currently a vast amount of evidence in the literature suggesting that matrix metalloproteinases (MMPs) and tissue inhibitors of metalloproteinases (TIMPs) are involved in the pathogenesis of inflammatory airways diseases, such as asthma and COPD. Despite this, the majority of reports only focus on single MMPs, often only in one model system. This study aimed to investigate the profile of an extensive range of MMP/TIMP levels in three different pre-clinical models of airways disease. These models each have a different and very distinct inflammatory profile, each exhibiting inflammatory characteristics that are similar to that observed in asthma or COPD. Since these models have their own characteristic pathophysiological phenotype, one would speculate that the MMP/TIMP expression profile would also be different.

**Methods:**

With the use of designed and purchased MMP/TIMP assays, investigation of rat MMP-2, 3, 7-14 and TIMP-1-4 mRNA expression was undertaken by Real Time PCR. The three rodent models of airways disease investigated were the endotoxin model, elastase model, and the antigen model.

**Results:**

Intriguingly, we demonstrated that despite the distinct inflammatory profile observed by each model, the MMP/TIMP expression profile is similar between the models, in that the same MMPs/TIMPs were observed to be generally increased or decreased in all three models. It could therefore be speculated that in a particular disease, it may be a complex network of MMPs, rather than an individual MMP, together with inflammatory cytokines and other mediators, that results in the distinct phenotype of inflammatory diseases, such as asthma and COPD.

**Conclusion:**

We believe our data may provide key information necessary to understand the role of various MMPs/TIMPs in different inflammatory airway diseases, and aid the development of more selective therapeutics without the side effect profile of current broad-spectrum MMP inhibitors.

## Background

Matrix metalloproteinases (MMPs) play a critical role in inflammatory airways diseases, such as chronic obstructive pulmonary disease (COPD) [1-4], and asthma [5-8]. However, the precise role of MMPs in inflammation still remains unclear although the role of this family of proteases has been studied extensively in pre-clinical models of airway inflammatory disease that share certain features of the human disease phenotype. Therefore, despite the vast literature implicating the involvement of these proteases in the pathogenesis of inflammatory diseases, many of these reports only focus on the role of one particular MMP, and often only in one model system. Hence, we were interested in investigating the profile of a large range of MMPs and their inhibitors, tissue inhibitors of metalloproteinases (TIMPs), in different inflammatory airways disease conditions modelled by three distinct pre-clinical models of inflammation. These three pre-clinical models: evoked by antigen, endotoxin and elastase, each exhibit their own distinct inflammatory characteristics that are similar to that observed in human airways disease, for example, increased eosinophils in asthma, and increased neutrophils and lymphomononuclear cells in inflammatory airways diseases, such as COPD. The antigen induced allergic airway inflammation model has been demonstrated to exhibit increased levels of eosinophils and inflammatory cytokines [9,10]. In addition, this model has also been demonstrated to have increased levels of p65:DNA binding, used as a marker of NF-κB pathway activation, and the antigen induced airway inflammation was observed to be responsive to steroid treatment. Our group has also demonstrated that this model exhibits a steroid insensitive early asthmatic response (EAR), and a steroid sensitive late asthmatic response (LAR). The endotoxin-driven model is predominantly neutrophilic in nature, and additionally differs from the antigen model because it is an innate response rather than an adaptive one. It has been shown to have increased levels of inflammatory cytokines and p65:DNA binding after stimulation, and we have also previously demonstrated the LPS induced inflammation to be sensitive to steroid treatment [11,12]. The third model we were interested in investigating in this study was the elastase induced experimental emphysema model, which has been demonstrated to exhibit an increase in lymphomononuclear cells and inflammatory cytokines [13]. Interestingly, the inflammation observed in the elastase model was steroid resistant, an aspect similar to that observed in emphysema/COPD. Furthermore, there was no increase in levels of p65:DNA binding at several selected time points after elastase treatment. In addition, this steroid resistant model exhibited aspects of airway remodelling, as average airspace area were increased, and emphysema-like changes in lung function were observed.

Since these three pre-clinical models each have different and very distinct inflammatory characteristics, one would speculate that the profile of MMPs and TIMPs involved may vary between these models. This study adopted the novel approach of elucidating the expression profile of a range of MMPs and TIMPs with the use of assays for TaqMan Real Time PCR, in these three distinct pre-clinical models of airways disease. We chose to use Real Time PCR, since there is a limited range of investigational techniques that are commercially available for the range of rat MMPs and TIMPs investigated in this study. We believe our data may provide key information necessary to understand the role of various MMPs and TIMPs in different inflammatory airway diseases, and aid the development of more selective therapeutics without the side effect profile of current broad-spectrum MMP inhibitors.

## Methods

Male Brown Norway rats (200-225 g), male Wistar rats (175-200 g) and male Sprague Dawley rats (260-300 g) were purchased from Harlan-Olac (Bicester, UK) and kept for at least 5 days before initiating experiments. Food and water were supplied *ad libitum*. UK Home Office guidelines for animal welfare based on the Animals (Scientific Procedures) Act 1986 were strictly observed.

Brown Norway rats were sensitised on days 0, 14 and 21 with ovalbumin (100 μg, i.p.) administered with aluminium hydroxide (100 mg, i.p.) and challenged with inhaled ovalbumin (10 g/l, 30 minutes) or saline aerosol on day 28, similar to that outlined by Underwood et al., 2002 [10]. For the time course studies, BAL fluid were obtained at various time points (2, 4, 6, 8, 12 and 24 hours), for analysis of cellular inflammation, biomarker levels by ELISA, and MMP-9 levels by zymography as outlined by McCluskie et al., 2004 [14]. Lung lobes were obtained to determine mRNA levels, as outlined by McCluskie et al., 2004 [14]. The effect of an IkappaB kinase-2 (IKK-2) inhibitor, TPCA-1 (2-[(aminocarbonyl)amino]-5-(4-fluorophenyl)-3-thiophenecarboxamide) and budesonide was investigated in this model. TPCA-1 (30 mg/kg, prepared in DMSO (2%), CremophorEL (10%) and ethanol (5%) in water) (donated by GlaxoSmithKline) or budesonide (3 mg/kg) (Sigma, UK) were orally dosed 2 hours before challenge, and 3, 8 and 12 hours after challenge. Budesonide, a commonly used steroid in man, was used as a positive control in these *in vivo *experiments, as it has previously been shown to inhibit LPS-induced neutrophilia in the rat [11]. This dosing regimen was used as it was found to give adequate compound exposure as assessed by pharmacokinetic studies and efficacy studies [9]. The dosing regimen for budesonide has been validated in our previous studies [15,11]. BAL fluid and lung lobes were taken 24 hours after challenge for analysis of cellular inflammation. The level of NF-κB pathway activation was determined on the lung tissue using an Active Motif kit which measures p65:DNA binding in accordance with manufactures instructions.

Wistar rats were challenged with aerosolised endotoxin free saline or LPS (0.3 mg/ml) for 30 minutes, as outlined by [12]. For time course studies, BAL fluid was obtained for analysis as described above, at various time points (1, 2, 4, 6, 8, 12 and 24 hours). For compound studies, TPCA-1 or budesonide was administered using the dosing regimen as above, 1 hour before challenge, and 2 hours after challenge. BAL fluid and lung lobes were taken 6 hours after challenge for analysis of cellular inflammation, and level of NF-κB pathway activation, as described above.

Using the elastase induced experimental emphysema model previously characterised by our group [13], BAL fluid and lung lobes were obtained for analysis as described above for time course studies, at 2, 6, 24, 48, 72, 96 and 168 hours. For compound studies, TPCA-1 or budesonide was administered using the dosing regimen as above, 1 hour prior and 6, 22, 30 and 46 hours post elastase insult. BAL fluid and lung lobes were taken 48 hours after challenge for analysis of cellular inflammation, and level of NF-κB pathway activation, as described above.

### MMP/TIMP mRNA levels by Real Time PCR

Total cellular RNA was isolated from all rat lung samples using Tri Reagent (Sigma-Aldrich), following manufacturer's instructions. RNA samples were reverse transcribed as outlined by [14]. Amplification and detection of MMPs 2, 3, 7-14 and TIMPs 1-4 mRNA was carried out in an ABI PRISM 7700 sequence detection system (Applied Biosystems), as outlined by [14], using designed, validated and optimised primers and TaqMan probes or validated pre-developed assays (Applied Biosystems) (Table 1). 18S rRNA levels were simultaneously measured to normalise for variations in sample loading. Due to the exponential nature of PCR, the delta ct (Δct) values were converted to a linear form, and written as 2^-Δct^. For graphing, 2^-ΔCt ^values were multiplied by 10^6 ^and shown as relative units. 2^-Δct ^values of less than 0.10 × 10^6 ^were assigned as 'below reliable detection limit' (BRDL).

**Table 1:** Table showing the sequences of designed and purchased rat primers and probes of MMPs investigated, the mRNA sequences used for the designs, and the conditions and optimised concentrations of each primer and probe.

**Assay**	**PUBMED Assession No**	**Assay Efficiency**		**Sequence**
**rat MMP-2**	NM_031054	**88.57%**	**Assay on Demand**	Applied Biosystems - Rn01538174_m1
				GATTCTGCCCAGAGACTGCTATGTC

**rat MMP-3**	NM_133523	**95.04%**	**Forward Primer**	5' CCT CTA TGG ACC TCC CAC AGA A 3'
			**Probe**	5' TAC CCA CCA AAT CTA ACT CTC TGG ACC CTG AG 3'
			**Reverse Primer**	5' CGA AGG ACA AAG CAG AGC TAC AC 3'

**rat MMP-7**	NM_012864	**104.02%**	**Forward Primer**	5' CGG CGG AGA TGC TCA CTT T 3'
			**Probe**	5' ACA GGA AGT TCA CTC CTG AGT CCT CAC CAT C 3'
			**Reverse Primer**	5' GCC AAG TTC ATG AGT GGC AAC 3'

**rat MMP-8**	NM_022221	**96.15%**	**Assay on Demand**	Applied Biosystems - Rn00573646_m1
				GAAAACTGCTGAGAATTACCTACGA

**rat MMP-9**	NM_031055	**99.79%**	**Forward Primer**	5' CCG AGA CCT GAA AAC CTC CAA 3'
			**Probe**	5' ACA CAC AGC TGG CAG AGG ATT ACC TGT ACC 3'
			**Reverse Primer**	GCT GCC CGA GTG TAA CCA TAG 3'

**rat MMP-10**	NM_133514	**94.89%**	**Forward Primer**	5' TCC TGG TGC TGC TGT GCT T 3'
			**Probe**	5' CCG ATC TGC TCA GCA TAT CCT CTG CAT 3'
			**Reverse Primer**	5' CTA GGT ATT GCT GAG CAA GAT CCA T 3'

**rat MMP-11**	NM_012980	**95.63%**	**Forward Primer**	5' CCT GTG GAT GCA GCT TTT GAG 3'
			**Probe**	5' CCA GAT TTG GTT CTT CCA AGG TGC TCA GTA C 3'
			**Reverse Primer**	5' GGG CCT AGG ACT GGC TTC TC 3'

**rat MMP-12**	NM_053963	**107.62%**	**Assay on**	Applied Biosystems - Rn00588640_m1
			**Demand**	TTCAAGGCACAAACCTGTTCCTTGT

**rat MMP-13**	XM_217083	**98.34%**	**Forward Primer**	5' CCA GTC TCT CTA TGG TCC AGG AGA T 3'
			**Probe**	5' AGA CCC CAA CCC TAA GCA CCC CAA AAC 3'
			**Reverse Primer**	5' TCG GAG ACT AGT AAT GGC ATC AAG 3'

**rat MMP-14**	NM_031056	**97.42%**	**Forward Primer**	5' CCG CCA TGC AAA GGT TCT A 3'
			**Probe**	5' CGA ATC GGC CTT GCC TGT CAC TTG 3'
			**Reverse Primer**	5' CGC CTC ATA GCC TTC ATC GT 3'

**rat TIMP-1**	NM_053819	**96.22%**	**Forward Primer**	5' CCA CCT TAT ACC AGC GTT ATG AGA 3'
			**Probe**	5' CGT CGA ATC CTT TGA GCA TCT TAG TCA TCT TG 3'
			**Reverse Primer**	5' CCG GAA ACC TGT GGC ATT T 3'

**rat TIMP-2**	NM_021989	**98.5%**	**Forward Primer**	5' GCT GGA CGT TGG AGG AAA GA 3'
			**Probe**	5' TCT CCT TCC GCC TTC CCT GCA ATT AGA TAT T 3'
			**Reverse Primer**	5' GCA CAA TAA AGT CAC AGA GGG TAA TG 3'

**rat TIMP-3**	NM_012886	**100.27%**	**Forward Primer**	5' GAA CGG AAG CGT GCA CAT G 3'
			**Probe**	5' CCG ACA TCG TGA TCC GGG CC 3'
			**Reverse Primer**	5' CCC TTC CTT CAC CAG CTT CTT 3'

**rat TIMP-4**	NM_080639	**97.27%**	**Forward Primer**	5' TGC AGA GGG AGA GCC TGA A 3'
			**Probe**	5' ACT GTG GCT GCC AAA TCA CCA CTT GC 3'
			**Reverse Primer**	5' GCC AGT CCG TCC AGA GAC A 3'

### Statistical analysis

Statistical significance was determined using an unpaired t test with each independent group compared to the vehicle control. If the variances of the two groups were significantly different then the Mann Whitney rank sum test was used. A p-value of less than 0.05 was taken as significant and denoted with *. All the values are expressed as mean ± s.e. mean of 6 observations.

## Results

### Cellular and biomarker inflammation in three different pre-clinical models of airways disease

**Figure 1:**
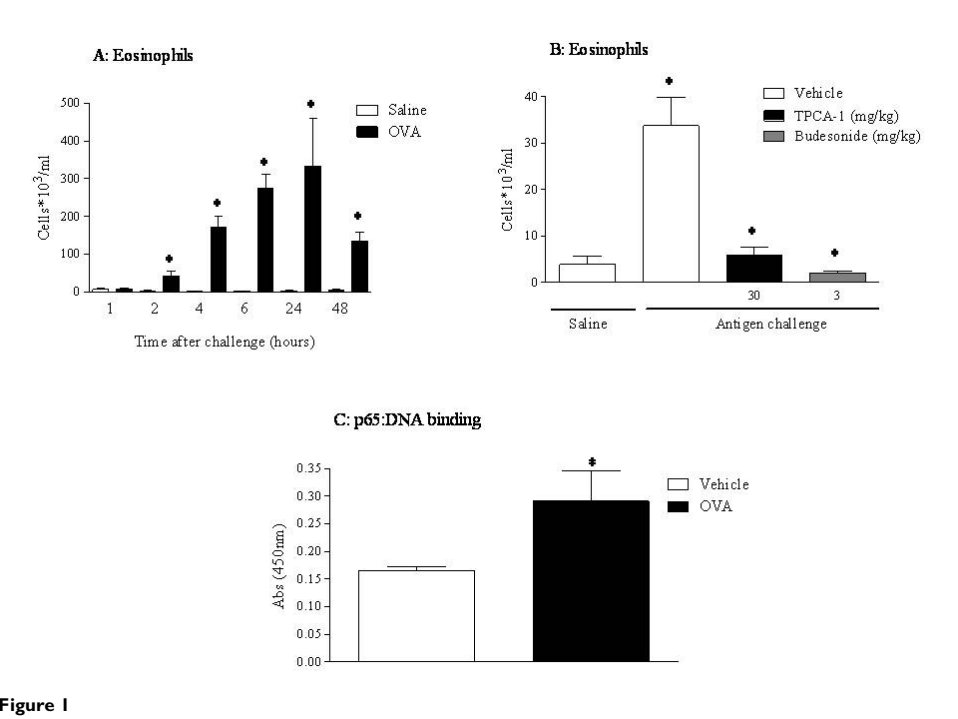
**Antigen induced airway inflammation model**. Rats were sensitised on days 0, 14 and 21 with ovalbumin (OVA) (100 μg, i.p.) administered with aluminium hydroxide (100 mg, i.p.) and challenged with inhaled OVA (10 g/l, 30 minutes) or saline aerosol on day 28. BAL samples were taken at each time point for determination of A: eosinophil levels (neutrophil and LMN levels are shown in Table 2). B: Effect of an IKK-2 inhibitor (TPCA-1) or budesonide on BAL eosinophil levels. C: Levels of p65:DNA binding in the lung tissue. Statistical significance was determined using an unpaired t test with each independent group compared to the vehicle control. If the variances of the two groups were significantly different then the Mann Whitney rank sum test was used. A p-value of less than 0.05 was taken as significant and denoted with *. All the values are expressed as mean ± s.e. mean of 6 observations.

**Figure 2:**
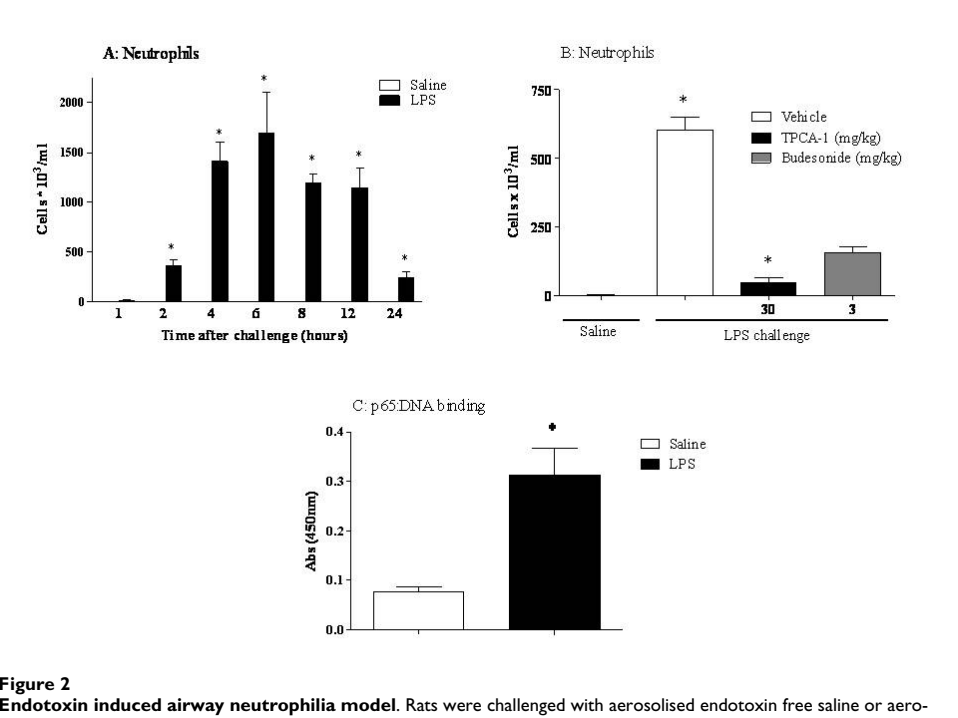
**Endotoxin induced airway neutrophilia model**. Rats were challenged with aerosolised endotoxin free saline or aerosolised LPS (0.3 mg/ml) for 30 minutes. BAL samples were taken at each time point for determination of A: neutrophil levels (eosinophils and LMN levels are shown in Table 2). B: Effect of an IKK-2 inhibitor (TPCA-1) or budesonide on BAL neutrophil levels. C: Levels of p65:DNA binding in the lung tissue. Statistical significance was determined using an unpaired t test with each independent group compared to the vehicle control. If the variances of the two groups were significantly different then the Mann Whitney rank sum test was used. A p-value of less than 0.05 was taken as significant and denoted with *. All the values are expressed as mean ± s.e. mean of 6 observations.

**Figure 3:**
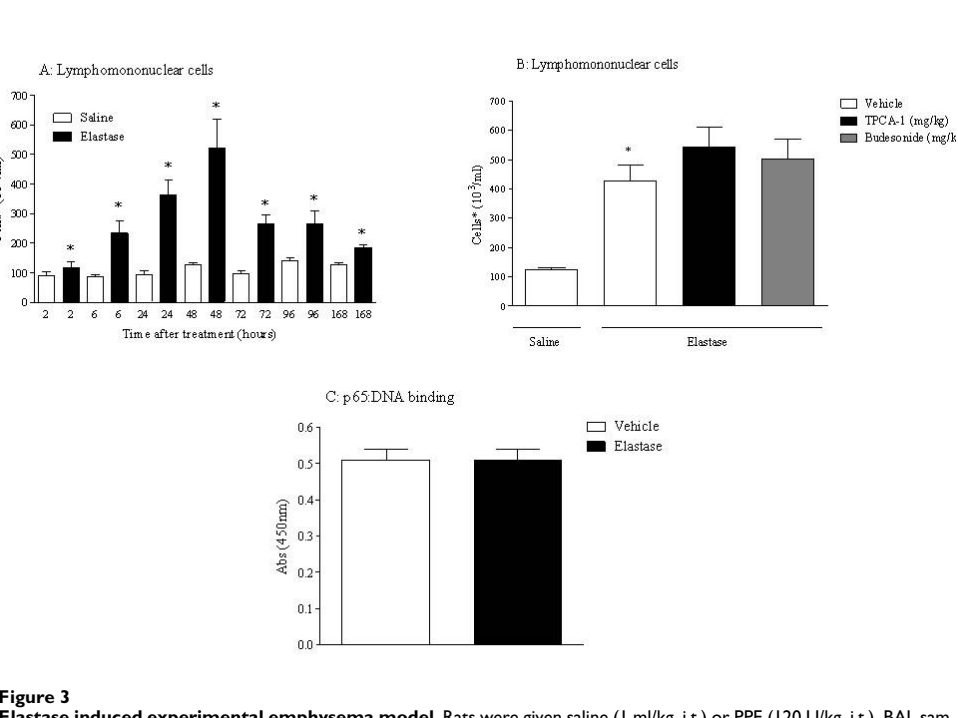
**Elastase induced experimental emphysema model**. Rats were given saline (1 ml/kg, i.t.) or PPE (120 U/kg, i.t.). BAL samples were taken at each time point for determination of A: LMN levels (neutrophil and eosinophil levels are shown in Table 2). B: Effect of an IKK-2 inhibitor (TPCA-1) or budesonide on BAL lymphomononuclear cell levels. C: Levels of p65:DNA binding in the lung tissue. Statistical significance was determined using an unpaired t test with each independent group compared to the vehicle control. If the variances of the two groups were significantly different then the Mann Whitney rank sum test was used. A p-value of less than 0.05 was taken as significant and denoted with *. All the values are expressed as mean ± s.e. mean of 6 observations.

The pre-clinical models of airways disease investigated have been evoked using a different stimuli: ovalbumin, LPS and elastase, and have previously been shown by our group to each exhibit characteristics that are similar to that observed in asthma [9,10], or COPD [13,12]. Figs. 1A, 2A, 3A and Table 2 show the inflammatory cell profiles observed in these models, with the antigen model mimicking allergic eosinophilia and neutrophilia; the endotoxin model displaying predominately innate neutrophilia, and the elastase-driven model featuring an increase in lymphomononuclear cells and neutrophils. Despite these three models each displaying an inflammatory profile, interestingly, the cellular inflammation could only be inhibited in the antigen model and the endotoxin model, after treatment with an IKK-2 inhibitor (TPCA-1) and budesonide, a steroid commonly used in the clinic to treat patients. These two compounds have previously been shown by our group to have no effect in the elastase model, further highlighting the fact that these three models each exhibit a different inflammatory profile (Fig. 1B, 2B and 3B). Moreover, when NF-κB pathway activation was investigated, the elastase model was observed to exhibit no increase in levels of p65:DNA binding after challenge, unlike the antigen model and the endotoxin model (Fig. 1C, 2C and 3C).

**Table 2:** BAL inflammatory cells in the three *in vivo *models of lung inflammation

**Antigen Model**			**LPS Model**			**Elastase Model**		
	**Neutrophils**	**LMN**		**Eosinophils**	**LMN**		**Neutrophils**	**Eosinophils**

**2 hrs Veh**	0.4 ± 0.3	173.8 ± 16.1	**1 hr Veh**	0.0 0.0	106.3 ± 20.1	**2 hrs Veh**	21.6 ± 5.9	1.1 ± 0.5

**2 hrs OVA**	11.7 ± 3.7	115.7 ± 18.8	**1 hr LPS**	6.1 ± 2.2	102.1 ± 10.7	**2 hrs ELA**	72.6 ± 15.8	0.0 ± 0.0

**4 hrs Veh**	2.8 ± 1.6	181.0 ± 16.0	**2 hrs Veh**	0.5 ± 0.3	155.7 ± 66.9	**6 hrs Veh**	80.3 ± 30.5	1.2 ± 0.4

**4 hrs OVA**	144.1 ± 60.1	99.0 ± 8.7	**2 hrs LPS**	31.9 ± 9.6	71.3 ± 8.9	**6 hrs ELA**	267 ± 86.7	1.9 ± 0.9

**6 hrs Veh**	1.5 ± 0.9	175.7 ± 21.3	**4 hrs Veh**	0.2 ± 0.2	80.7 ± 16.6	**24 hrs Veh**	46.8 ± 22.4	0.9 ± 0.4

**6 hrs OVA**	977.1 ± 300.9	150.7 ± 25.9	**4 hrs LPS**	95.1 ± 24.5	80.0 ± 18.5	**24 hrs ELA**	382.1 ± 77.4	4.3 ± 2.5

**8 hrs Veh**	0.2 ± 0.2	173.9 ± 12.3	**6 hrs Veh**	0.0 ± 0.0	151.8 ± 37.8	**48 hrs Veh**	8.6 ± 2.3	0.0 ± 0.0

**8 hrs OVA**	1324.5 ± 293.2	178.1 ± 23.0	**6 hrs LPS**	110.0 ± 28.3	112.1 ± 10.5	**48 hrs ELA**	529.4 ± 174.5	4.3 ± 4.3

**12 hrs Veh**	1.6 ± 0.6	176.4 ± 31.9	**8 hrs Veh**	0.0 ± 0.0	141.5 ± 31.5	**72 hrs Veh**	1.1 ± 0.6	0.5 ± 0.2

**12 hrs OVA**	998.7 ± 312.2	195.2 ± 27.0	**8 hrs LPS**	80.6 ± 11.2	139.3 ± 11.7	**72 hrs ELA**	83.2 ± 9.1	0.6 ± 0.6

**24 hrs Veh**	0.7 ± 0.4	171.2 ± 15.9	**12 hrs Veh**	0.1 ± 0.1	100.5 ± 13.9	**96 hrs Veh**	1.9 ± 1.4	0.3 ± 0.3

**24 hrs OVA**	317.4 ± 141.3	390.1 ± 85.9	**12 hrs LPS**	41.3 ± 6.5	213.3 ± 10.4	**96 hrs ELA**	18.1 ± 8.4	1.7 ± 0.9

			**24 hrs Veh**	0.1 ± 0.1	98.8 ± 15.9	**168 hrs Veh**	17.2 ± 14.1	0.4 ± 0.4

			**24 hrs LPS**	7.9 ± 2.0	75.5 ± 12.9	**168 hrs ELA**	0.6 ± 0.4	0.3 ± 0.3

### Determination of MMP/TIMP mRNA levels in three distinct in vivo models of airways disease

#### In vivo model of antigen induced airway inflammation

**Figure 4:**
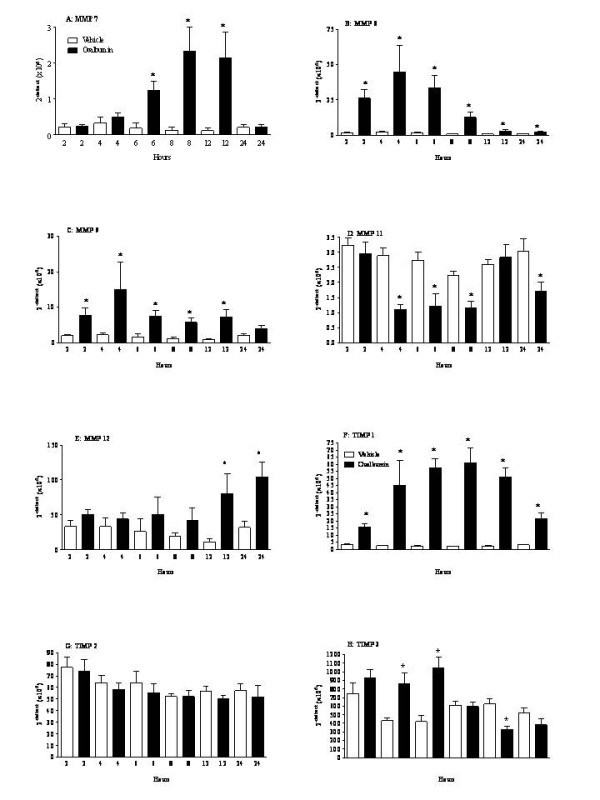
**MMP mRNA levels in the *in vivo *model of antigen induced airway inflammation**. Rats were sensitised on days 0, 14 and 21 with ovalbumin (OVA) (100 μg, i.p.) administered with aluminium hydroxide (100 mg, i.p.) and challenged with inhaled OVA (10 g/l, 30 minutes) or saline aerosol on day 28. Rats were sacrificed with an overdose of sodium pentobarbitone, and lung lobes were obtained for mRNA levels. MMP mRNA levels were determined by Real Time PCR (A: MMP-7; B: MMP-8; C: MMP-9; D: MMP-11; E: MMP-12; F: TIMP-1; G: TIMP-2 and H: TIMP-3). Table 3 shows the data for the remaining MMPs/TIMPs. Data were deemed to be BRDL, if the value was less than 0.1. MMP-10 and 13 were BRDL. Where the levels in the time-matched vehicle controls were BRDL, statistical significance could not be determined. Statistical significance was determined using an unpaired t test with each independent group compared to the time matched vehicle control. If the variances of the two groups were significantly different then the Mann Whitney rank sum test was used. A p-value of less than 0.05 was taken as significant and denoted with *. All the values are expressed as mean ± s.e. mean of 6 observations.

In the antigen induced airway inflammation model, which has been shown to exhibit aspects similar to the inflammation observed in asthma, MMP-7 mRNA levels were found to be increased as early as 4 hours after ovalbumin challenge (Fig. 4A). MMP-8 and 9 mRNA levels were found to have a similar profile, where levels were significantly increased after ovalbumin (Fig. 4B and 4C). Ovalbumin challenge was also demonstrated to increase MMP-12 and 14 mRNA levels (Fig. 4E and Table 3 respectively). Interestingly, MMP-11 mRNA levels were observed to decrease after challenge (Fig. 4D), and MMP-3, 10 and 13 mRNA levels were either BRDL or very low (Table 3). Basal MMP-2 mRNA levels were measured at all the time points, which appeared not to change after antigen challenge, except at the 24 hour time point where a significant decrease was observed after antigen challenge (Table 3). Surprisingly, a significant increase in TIMP-1 and TIMP-3 mRNA levels were observed after treatment (Fig. 4F and 4H), whereas a general decrease was observed in TIMP-2 mRNA levels (Fig. 4G). TIMP-4 mRNA levels were observed to be low and ovalbumin challenge appeared to decrease these levels (Table 3).

**Table 3:** MMP mRNA levels in the *in vivo *model of antigen induced airway inflammation.

	**MMP-2**	**MMP-3**	**MMP-14**	**TIMP-4**
**2 hrs Vehicle**	72.1 ± 10.2	BRDL	18.6 ± 2.3	0.3 ± 0.0

**2 hrs Ovalbumin**	84.3 ± 6.9	BRDL	33.8 ± 1.7 *	0.2 ± 0.1

**4 hrs Vehicle**	77.1 ± 11.7	BRDL	24.1 ± 4.0	0.3 ± 0.1

**4 hrs Ovalbumin**	71.3 ± 2.5	0.1 ± 0.0	28.7 ± 2.3	0.2 ± 0.0

**6 hrs Vehicle**	58.3 ± 11.5	BRDL	21.7 ± 3.6	0.3 ± 0.0

**6 hrs Ovalbumin**	73.0 ± 6.9	BRDL	40.9 ± 7.5 *	0.2 ± 0.1

**8 hrs Vehicle**	52.7 ± 3.7	BRDL	18.5 ± 2.5	0.6 ± 0.1

**8 hrs Ovalbumin**	64.1 ± 5	BRDL	43.5 ± 7.4 *	0.2 ± 0.1 *

**12 hrs Vehicle**	82.0 ± 9.7	0.2 ± 0.0	17.0 ± 3.1	0.6 ± 0.1

**12 hrs Ovalbumin**	71.3 ± 3.9	BRDL	50.7 ± 5.7 *	0.2 ± 0.0 *

**24 hrs Vehicle**	75.9 ± 5.5	0.1 ± 0.0	22.5 ± 2.1	0.3 ± 0.1

**24 hrs Ovalbumin**	48.3 ± 8.5 *	0.2 ± 0.0	42.0 ± 8.8	0.2 ± 0.1 *

A p-value of less than 0.05 was taken as significant and denoted with *. MMP-10 and 13 were BRDL.

#### In vivo model of LPS induced neutrophilia

**Figure 5:**
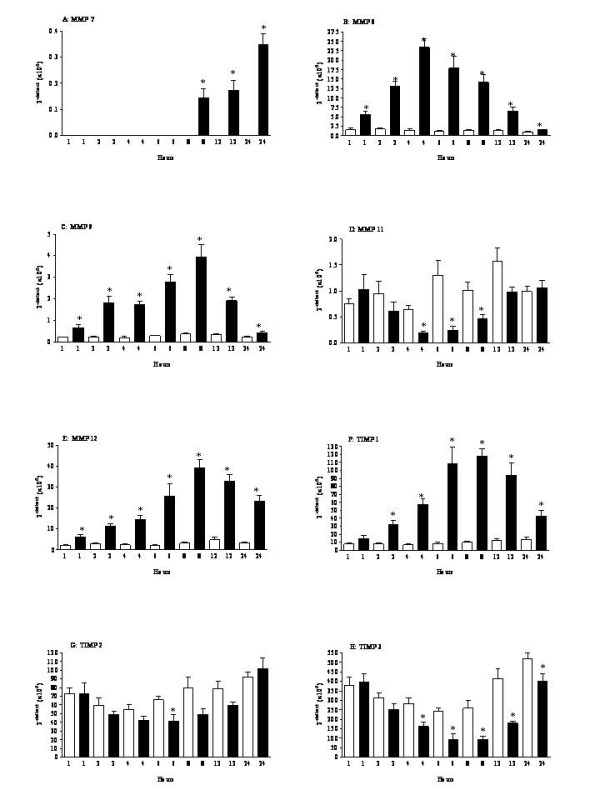
**MMP mRNA levels in the *in vivo *model of LPS induced neutrophilic inflammation**. Rats were challenged with aerosolised endotoxin free saline or aerosolised LPS (0.3 mg/ml) for 30 minutes, and were sacrificed with an overdose of sodium pentobarbitone, and lung lobes were obtained for mRNA levels. MMP mRNA levels were determined by Real Time PCR (A: MMP-7; B: MMP-8; C: MMP-9; D: MMP-11; E: MMP-12; F: TIMP-1; G: TIMP-2 and H: TIMP-3). All the values are expressed as mean ± s.e. mean of 6 observations. Data in the graphs are expressed as relative units. Table 4 shows the data for the remaining MMPs/TIMPs. Data were deemed to be BRDL, if the value was less than 0.1. MMP-3, 10 and 13 were BRDL. Where the levels in the time-matched vehicle controls were BRDL, statistical significance could not be determined. Statistical significance was determined using an unpaired t test with each independent group compared to the time matched vehicle control. If the variances of the two groups were significantly different then the Mann Whitney rank sum test was used. A p-value of less than 0.05 was taken as significant and denoted with *. All the values are expressed as mean ± s.e. mean of 6 observations.

In the rat model of LPS induced airway neutrophilia, MMP-7 mRNA levels were detected from 8 hours after LPS challenge (Fig. 5A). The profile of MMP-8, 9 and 12 mRNA levels appear to be different to MMP-7, where these levels were found to be significantly increased with time after LPS challenge (Fig. 5B, C and 5E). Interestingly, similar to the antigen model, MMP-11 mRNA levels were also found to be significantly decreased after challenge (Fig. 5D). There was no significant difference in MMP-14 mRNA levels after LPS challenge (Table 4). TIMP-1 mRNA levels had a similar profile to MMP-8, 9 and 12, where a significant increase in mRNA level was observed with time (Fig. 5F). TIMP-2 and 3 mRNA levels were observed to be significantly decreased at some of the time points after LPS challenge (Fig. 5G and 5H). Similar to the antigen model, MMP-2, 3, 10, 13 and TIMP-4 mRNA levels were either BRDL, low or no significant difference was observed between vehicle and treated groups (Table 4).

**Table 4:** MMP mRNA levels in the *in vivo *model of LPS induced neutrophilic inflammation.

	**MMP-2**	**MMP-14**	**TIMP-4**
**1 hr Vehicle**	90.3 ± 8.3	10.8 ± 0.8	0.3 ± 0.1

**1 hr LPS**	136.4 ± 35.2	13.2 ± 2.9	0.3 ± 0.1

**2 hrs Vehicle**	64.6 ± 1.7	9.7 ± 0.3	0.3 ± 0.04

**2 hrs LPS**	60.4 ± 7.8	10.5 ± 1.5	0.2 ± 0.1

**4 hrs Vehicle**	82.8 ± 6.8	11.1 ± 1.0	0.2 ± 0.1

**4 hrs LPS**	89.7 ± 7.7	11.8 ± 1.5	BRDL

**6 hrs Vehicle**	60.8 ± 13.4	10.4 ± 0.5	0.2 ± 0.04

**6 hrs LPS**	82.8 ± 10.7	14.1 ± 2.2	BRDL

**8 hrs Vehicle**	107.9 ± 22.4	19.5 ± 3.7	0.4 ± 0.1

**8 hrs LPS**	77.5 ± 12.1	23.9 ± 2.6	0.2 ± 0.1

**12 hrs Vehicle**	110.6 ± 13.0	22.9 ± 3.4	0.2 ± 0.03

**12 hrs LPS**	79.3 ± 6.8	21.0 ± 1.3	0.3 ± 0.1

**24 hrs Vehicle**	97.3 ± 13.8	20.8 ± 2.3	0.4 ± 0.1

**24 hrs LPS**	80.9 ± 3.9 ±	23.6 ± 1.6	0.3 ± 0.1

A p-value of less than 0.05 was taken as significant and denoted with *. MMP-3, 10 and 13 were BRDL.

#### In vivo model of elastase driven experimental emphysema

**Figure 6:**
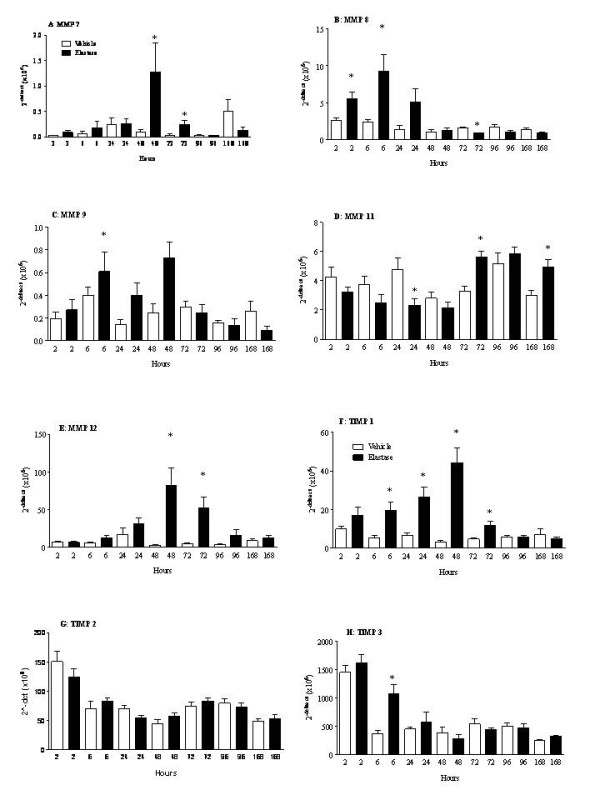
**MMP mRNA levels in the *in vivo *model of elastase driven experimental emphysema**. Rats were given saline (1 ml/kg, i.t.) or PPE (120 U/kg, i.t.) and were sacrificed with an overdose of sodium pentobarbitone, and lung lobes were obtained for mRNA levels. MMP mRNA levels were determined by Real Time PCR (A: MMP-7; B: MMP-8; C: MMP-9; D: MMP-11; E: MMP-12; F: TIMP-1; G: TIMP-2 and H: TIMP-3). Table 5 shows the data for the remaining MMPs/TIMPs. Data were deemed to be BRDL, if the value was less than 0.1. MMP-3, 10 and 13 were BRDL. Where the levels in the time-matched vehicle controls were BRDL, statistical significance could not be determined. Statistical significance was determined using an unpaired t test with each independent group compared to the time matched vehicle control. If the variances of the two groups were significantly different then the Mann Whitney rank sum test was used. A p-value of less than 0.05 was taken as significant and denoted with *. All the values are expressed as mean ± s.e. mean of 6 observations.

MMP-8 mRNA levels were found to be increased at the earlier time points after elastase treatment (Fig. 6B). The profiles of MMP-7, 9, 12, 14 and TIMP-1 mRNA levels were similar to each other in this model, as mRNA levels were found to be highest 48 hours after elastase treatment (Fig. 6A, C, E, Table 5 and Fig. 6F, respectively). Similar to the antigen model and the endotoxin model, MMP-11 mRNA levels were also found to be decreased after treatment (Fig. 6D). No significant difference was observed in TIMP-2 mRNA levels after elastase insult (Fig. 6G). TIMP-3 mRNA levels were found to be highly expressed in all three pre-clinical models investigated, and were observed to be significantly increased, 6 hours after elastase treatment (Fig. 6H). Similar to the other two models, MMP-2, 3, 10, 13 and TIMP-4 mRNA levels were also found to be BRDL, low in all the groups, or no significant difference was observed between vehicle and treated groups (Table 5).

**Table 5:** MMP mRNA levels in the *in vivo *model of elastase driven experimental emphysema.

	**MMP-2**	**MMP-14**	**TIMP-4**
**2 hrs Vehicle**	162.0 ± 14.7	38.4 ± 3.2	1.2 ± 0.2

**2 hrs Elastase**	168.4 ± 20.4	36.0 ± 2.5	0.7 ± 0.2

**6 hrs Vehicle**	76.9 ± 8.3	25.0 ± 3.3	0.5 ± 0.1

**6 hrs Elastase**	86.6 ± 8.3	36.6 ± 4.9	0.7 ± 0.1

**24 hrs Vehicle**	84.9 ± 10.9	18.3 ± 3.6	0.4 ± 0.04

**24 hrs Elastase**	59.7 ± 7.7	18.6 ± 2.4	0.2 ± 0.1 *

**48 hrs Vehicle**	70.8 ± 16.6	9.4 ± 2.0	0.5 ± 0.1

**48 hrs Elastase**	60.6 ± 4.1	42.2 ± 6.1 *	0.2 ± 0.1 *

**72 hrs Vehicle**	90.7 ± 9.3	15.5 ± 1.7	0.5 ± 0.1

**72 hrs Elastase**	74.7 ± 6.2	36.0 ± 5.7 *	0.3 ± 0.1

**96 hrs Vehicle**	95.6 ± 9.3	17.0 ± 2.4	0.6 ± 0.1

**96 hrs Elastase**	100.9 ± 11.1	23.5 ± 4.7	0.4 ± 0.05

**168 hrs Vehicle**	61.9 ± 5.9	16.8 ± 3.2	0.3 ± 0.1

**168 hrs Elastase**	66.0 ± 8.2	28.8 ± 5.2	0.2 ± 0.1

A p-value of less than 0.05 was taken as significant and denoted with *. MMP-3, 10 and 13 were BRDL.

### Determination of MMP-9 levels in three distinct in vivo models of airways disease

**Figure 7:**
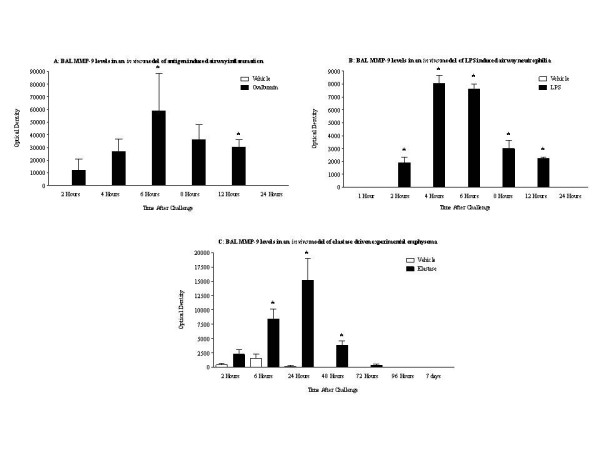
**BAL MMP-9 levels in three *in vivo *models of lung inflammation by zymography**. A: BAL MMP-9 levels in the antigen model: rats were sensitised on days 0, 14 and 21 with ovalbumin (OVA) (100 μg, i.p.) administered with aluminium hydroxide (100 mg, i.p.) and challenged with inhaled OVA (10 g/l, 30 minutes) or saline aerosol on day 28. B: BAL MMP-9 levels in the LPS model: rats were challenged with aerosolised endotoxin free saline or aerosolised LPS (0.3 mg/ml) for 30 minutes. C: BAL MMP-9 levels in the elastase model: rats were given saline (1 ml/kg, i.t.) or PPE (120 U/kg, i.t.). All rats were sacrificed with an overdose of sodium pentobarbitone, and BAL was taken for MMP-9 analysis by zymography. Statistical significance was determined using an unpaired t test with each independent group compared to the time matched vehicle control. If the variances of the two groups were significantly different then the Mann Whitney rank sum test was used. A p-value of less than 0.05 was taken as significant and denoted with *. All the values are expressed as mean ± s.e. mean of 6 observations.

MMP-9 levels were determined in the BAL from the antigen model, LPS model and the elastase model, using zymography. BAL MMP 9 levels were observed to be increased with time after ovalbumin, LPS or elastase insult (Fig. 7A, B and 7C respectively).

## Discussion

There is a wealth of information in the literature speculating that MMPs may play a critical role in inflammatory diseases, such as asthma and COPD. This is the first study in the literature that compares the inflammatory profiles in three distinct pre-clinical models, each evoked by a different stimulus to mimic some of the inflammatory characteristics that are observed in asthma or COPD. The first part of this study compares the profile of cellular inflammation and inflammatory cytokines between the three models. The data show that these three models each have distinct inflammatory characteristics which are exhibited in disease, for example, increased eosinophils in asthma or increased neutrophils and lymphomononuclear cells in inflammatory airways diseases, such as COPD. In addition, the inflammation in both the antigen model and the endotoxin model were observed to be steroid-sensitive and involve the IKK/NF-κB pathway, whereas the elastase model, a model that we have previously demonstrated to have structural lung changes, was shown to be steroid resistant and without involvement of the IKK/NF-κB pathway. This first part of the study demonstrated that the three pre-clinical models investigated each have a different inflammatory profile, and since many reports only focus on the role of one particular MMP, and often only in one model system, we were interested in comparing the MMP/TIMP mRNA expression profiles between these different models. To enable this, we used designed and purchased primers and probes for TaqMan Real Time PCR.

Interestingly, our data demonstrated that although the three models of airways disease each have a very different and distinct inflammatory profile, the expression profile of lung MMPs 2, 7 - 10, 12 - 14, TIMP-1 and 4 mRNA levels were similar in each model. We chose to use Real Time PCR as there is a limited range of investigational techniques that are commercially available for the range of rat MMPs and TIMPs investigated in this study. To date, the range of rat MMP and TIMP ELISAs for measuring MMPs/TIMPs at the protein level remain very limited. One of the available techniques that has been widely used by researchers to investigate MMP-9 at the protein level, is zymography. This technique uses the activity of MMP-9 to assess levels of the mediator. In our study, the amounts of MMP-9 measured with zymography appear to temporally correlate with MMP-9 mRNA levels in all three models. This would suggest that the mRNA profiling data established in this manuscript is likely to be indicative of the amounts of the same target at the protein level. However, it should be noted that the profile of the different MMP/TIMP mRNA levels obtained in this study may not be equivalent to the activity status of the proteins investigated, since MMPs are regulated at various intra- and extracellular levels. Despite this, the data obtained from this study provides very useful information about the extensive range of MMPs and TIMPs investigated at the molecular level, where tools are limited for investigation at the protein level. To date, there does not appear to be any information in the literature on the profile of the extensive range of MMPs and TIMPs investigated in this study in these pre-clinical models of inflammation.

Three different strains of rats were used for the three different models since previous historical work performed by our group used these strains in the development of these "disease" models. An investigation of the MMP and TIMP mRNA expression profile in the naïve rat lungs of the three strains of rats investigated appeared to be similar, suggesting that these strains were comparable (data not shown). However, the differences that could exist between the three different strains cannot be completely ruled out.

It is believed that matrix remodelling is the result, in part, of a shift in the balance between active MMPs versus TIMPs, and it is thought that coordinated regulation of these proteases and anti-proteases is required to maintain tissue architecture [16]. Interestingly, this study demonstrated not only an increase in proteases, but also an increase in mRNA levels of TIMP-1, an anti-protease in inflammatory conditions. However, there is evidence in the literature suggesting that an over-expression of anti-protease could be due to the host's response to an increase in MMP, in an attempt to control MMP activity and retain extra cellular matrix integrity [17], which could possibly explain the increased TIMP-1 observed. There has also been evidence in the literature of elevated TIMP-1 levels, when investigated in the sputum of COPD patients [18,19]. The study by Mercer *et al *[19] demonstrated TIMP-1 levels to be increased in the sputum of stable COPD patients prior to exacerbations, but during exacerbations, MMP-9 levels were significantly increased, so tipping the balance in favour of MMP-9. Conversely, the profiles of TIMP-2, 3 and 4 mRNA levels were observed to be decreased overall in the models investigated, except for TIMP-3 levels in the elastase model.

## Conclusion

The findings of this study show that a range of MMPs are upregulated in the three different models investigated. Despite the fact that the kinetics of some of the MMP/TIMP expression were different, the overall MMP/TIMP profile is surprisingly similar between the three different pre-clinical models of airways disease, in that the same MMPs/TIMPs were generally increased or decreased in all three models. It could therefore be speculated that in a particular disease, it maybe a complex network of MMPs, rather than an individual MMP, together with inflammatory cytokines and other mediators, that results in the distinct phenotype of inflammatory diseases, such as asthma and COPD. To our knowledge, this study is the first to investigate such an extensive range of MMPs and TIMPs in a range of different models of inflammation. Overall, we believe this exciting data may provide very useful information necessary to understand the role of various MMPs and TIMPs in inflammatory airway diseases. Information regarding the specific pattern of MMPs/TIMPs involved in "disease" conditions may be useful for the development of novel therapeutics without the side effect profile of non-selective, broad-spectrum MMP inhibitors.

## List of abbreviations

OVA: Ovalbumin; BAL: Bronchoalveolar lavage; COPD: Chronic obstructive pulmonary disease; EAR: Early asthmatic response; ELA: Elastase; ELISA: Enzyme-linked immunosorbant assay; LAR: Late asthmatic response; LPS: Lipopolysaccharide; MMP: Matrix metalloproteinase; NF-κB: Nuclear factor kappa B; TIMP: Tissue inhibitor of metalloproteinase

## Competing interests

The authors declare that they have no competing interests.

## Authors' contributions

SW participated in the design of the study, performed the molecular analysis of the samples, data analysis, and drafted the manuscript. MAB performed the in vivo studies. MGB and MAB conceived the study and participated in the design of the study. All authors have read and approved the final manuscript.
